# Supplementary material for: Biallelic Loss of Function Mutation in Sodium Channel Gene SCN10A in an Autism Spectrum Disorder Trio from Pakistan
Source: Genes (Basel). 2022 Sep 11;13(9):1633. doi: 10.3390/genes13091633 (PMC9498319; doi:10.3390/genes13091633)
Supplement: Supplementary file 1 [file genes-13-01633-s001.zip › genes-1873279-supplementary.pdf]

Supplementary Table S1: filtered variants identified in SMPA19: All variants were validated by IGV (Integrated Genomics Viewer; Broad Institute) and by Sanger sequencing. Allele frequencies are from gnomAD v2.1.1 (www. https://gnomad.broadinstitute.org/; accessed Aug 2022). CADD Phred-like scores: https://cadd.gs.washington.edu/; M-CAP: PMID 27776117. T=tolerated; D=deleterious; B=benign; Poss D= possibly damaging.

Variant	Chr	Coordinates (hg19)	Ref.	Alt.	Gene	Variant Type	cDNA/Amino Acid Change	gnomAD All Freq.	gnomAD S. Asian Freq.	Polyphen2 HumVar	Mutation Taster	CADD/PHRED score (Prediction)
1	1	158576988	T	C	OR10Z1	Nonsynonymous	NM_001004478.2:exon1:c.760T>C:p.Cys254Arg	7.57E-05	6.21E-04	Poss D	D	24.3 (D)
2	3	38802185	C	A	**SCN10A**	Stopgain	NM_001293306.2: exon7: c.937G>T; p. Gly313*	3.98E-06	3.27E-05	-	D	39 (D)
3	3	47324525	G	A	KLHL18	Nonsynonymous	NM_025010.3:exon1:c.70G>A:p.Gly24Ser	1.32E-05	0	B	D	24.1 (D)
4	7	73922462	G	A	GTF2IRD1	Nonsynonymous	NM_001199207:exon2:c.52G>A:p.Asp18Asn	8.04E-06	3.27E-05	Poss D	D	23.6 (D)
5	X	140993480	A	G	MAGEC1	Nonsynonymous	NM_005462.4:exon4:c.290A>G:p.Gln97Arg	2.73E-05	2.62E-04	B	T	10.26 (T)

Variant 1 is in OR10Z1, which encodes an olfactory receptor, and can be excluded as a candidate. Variant 5, in MAGEC1, is a missense variant that doesn't pass the set criteria for predicted deleteriousness, and for Variants 3 and 4 the predictions are mixed. On the other hand, Variant 2, a stop gain in SCN10A, which has previously been reported in connection with ASD, is clearly a convincing candidate.

Variant Filtering:

As described in Harripaul et al. MedRXIV. Briefly, variants were compared against known dbSNP variants, and a convolutional neural network was trained to filter out poor calls based on several metrics (Van de Auwera et al, 2013), and minor allele frequency (MAF) in gnomAD (https://gnomad.broadinstitute.org/) of greater than 1×10^{-3} for autosomal recessive, or 1×10^{-5} for autosomal dominant/*de novo*, with a preference for variants that had no homozygotes in the gnomAD non-Neuro Cohort. For autosomal recessive variants, as the MAF used was on the conservative side, reanalysis was also performed using less stringent values (1×10^{-3}, 1×10^{-2}), to see if any variants in known ASD/ID genes were missed under the stringent analysis.

Van der Auwera GA, Carneiro MO, Hartl C, Poplin R, Del Angel G, Levy-Moonshine A, et al. From FastQ data to high confidence variant calls: the Genome Analysis Toolkit best practices pipeline. Curr Protoc Bioinforma. 2013;43:11.10.1-11.10.33.
